# Graft preservation in heart transplantation: current approaches

**DOI:** 10.3389/fcvm.2023.1253579

**Published:** 2023-08-11

**Authors:** Andrea Lechiancole, Sandro Sponga, Giovanni Benedetti, Arianna Semeraro, Giorgio Guzzi, Cristian Daffarra, Matteo Meneguzzi, Chiara Nalli, Daniela Piani, Marilyn Bressan, Ugolino Livi, Igor Vendramin

**Affiliations:** ^1^ Cardiothoracic Department, Azienda Sanitaria Universitaria Friuli Centrale, University Hospital of Udine, Udine, Italy; ^2^ Department of Medicine, University of Udine, Udine, Italy

**Keywords:** heart transplantation, donor organ preservation, normothermic *ex situ* perfusion, hypothermic perfusion, graft preservation

## Abstract

Heart transplantation (HTx) represents the current best surgical treatment for patients affected by end-stage heart failure. However, with the improvement of medical and interventional therapies, the population of HTx candidates is increasingly old and at high-risk for mortality and complications. Moreover, the use of “extended donor criteria” to deal with the shortage of donors could increase the risk of worse outcomes after HTx. In this setting, the strategy of donor organ preservation could significantly affect HTx results. The most widely used technique for donor organ preservation is static cold storage in ice. New techniques that are clinically being used for donor heart preservation include static controlled hypothermia and machine perfusion (MP) systems. Controlled hypothermia allows for a monitored cold storage between 4°C and 8°C. This simple technique seems to better preserve the donor heart when compared to ice, probably avoiding tissue injury due to sub-zero °C temperatures. MP platforms are divided in normothermic and hypothermic, and continuously perfuse the donor heart, reducing ischemic time, a well-known independent risk factor for mortality after HTx. Also, normothermic MP permits to evaluate marginal donor grafts, and could represent a safe and effective technique to expand the available donor pool. However, despite the increasing number of donor hearts preserved with these new approaches, whether these techniques could be considered superior to traditional CS still represents a matter of debate. The aim of this review is to summarize and critically assess the available clinical data on donor heart preservation strategies employed for HTx.

## 1. Introduction

Heart transplantation (HTx) is the current gold standard surgical treatment for end-stage heart failure. However, despite improvement in the management of HTx recipients, the rate of primary graft dysfunction (PGD) continues to be relatively high, being a severe complication that still represents the leading cause of 30-day mortality after HTx (1–3). A recent national study from Sing et al. reported an overall incidence of PGD after HTx of 36%, with moderate-to-severe PGD rate of 32% (4). The interaction of donor, recipient and procedural variables has been shown to predispose to this life-threatening complication.

Continuous improvements of medical and interventional therapies, as well as the wide employment of mechanical circulatory support supports (MCS), have allowed an increasingly old population with multiple comorbidities to be considered as HTx candidates. On the other hand, the use of extended donor criteria to face graft shortage has increased the risk of worse outcomes after HTx. Therefore, in this setting, the strategy of donor organ preservation could play a central role in improving HTx recipient outcomes and in preventing PGD.

The use of static cold storage (SCS) for donor graft preservation, aims to stabilize biological tissues by influencing metabolic pathways. Such strategy slows the cellular and extracellular biochemical processes that are responsible for organ degradation during ischemic storage, thus extending a safe storage time up to several hours. On the other hand, machine perfusion (MP) systems permit to continuously perfuse the coronary arteries, reducing ischemic time and potentially mitigate the deleterious effects of ischemia/reperfusion injury. Despite the increasing number of donor hearts preserved with MP, whether MP could be considered superior to traditional CS still represents a matter of debate.

The aim of this paper is to summarize and critically assess the available clinical data on the donor heart preservation strategies currently employed for HTx.

## 2. Static cold storage

Employment of SCS aims to stabilize biological tissues by slowing the cellular and extracellular biochemical processes that are responsible for organ degradation during ischemic storage, thus extending the safe storage time. The temperature dependence of chemical reaction rates follows the “Arrhenius equation”, used to describe the temperature-depending metabolic changes: for every 10°C reduction of temperature below the physiological temperature the metabolic rate for living biological tissues reduces by 50%. Hence, cold storage slows but does not completely arrest cellular metabolism. Consequently, progressive ischemic injury is an inevitable consequence of prolonged SCS and the results of HTx are suboptimal when graft ischemic time is greater than 6 h.

### 2.1. Cold solution and ice

The traditional ice-cold SCS remains the most commonly used technique for donor graft preservation, being both user friendly and cost-effective. In brief, after the donor heart is retrieved, it is placed into a sterile bag filled with 1,000 ml of preservation saline solution at 4°C which is then sealed into a second bag containing 1,000 ml of cold solution, and eventually in a third bag. Then, the heart is placed in a rigid sterile container filled with cold solution which is sealed and inserted into a cooler filled with ice for transportation.

Using conventional ice-cold SCS, prolonged ischemic time is known to be an independent risk factor for PGD and mortality after HTx (5–7). Moreover, the negative impact of graft ischemic time is considerably influenced by other donor characteristics, as age, left ventricular hypertrophy, mild-to-moderate coronary artery disease and catecholamine support (7).

As reported by the ISHLT Consensus Statement on donor heart and lung procurement (8), the ideal donor graft temperature during storage should probably be kept between 5°C and 10°C. In fact, freezing of any part of the heart is undesirable because freezing and subsequent thawing may cause tissue damage potentially responsible for PGD (9). Indeed, possible freeze injury was detected in 7% of autopsies done on patients deceased for clinically diagnosed PGD (1).

### 2.2. Controlled hypothermia with Paragonix SherpaPak cardiac transport system

The Paragonix SherpaPak™ cardiac transport system (PSP) is able to guarantee a constant, homogeneous and controlled temperature of the donor heart between 4°C and 8°C, thus minimizing tissue injury due to ice-cold temperature exposure.

**Figure 1:**
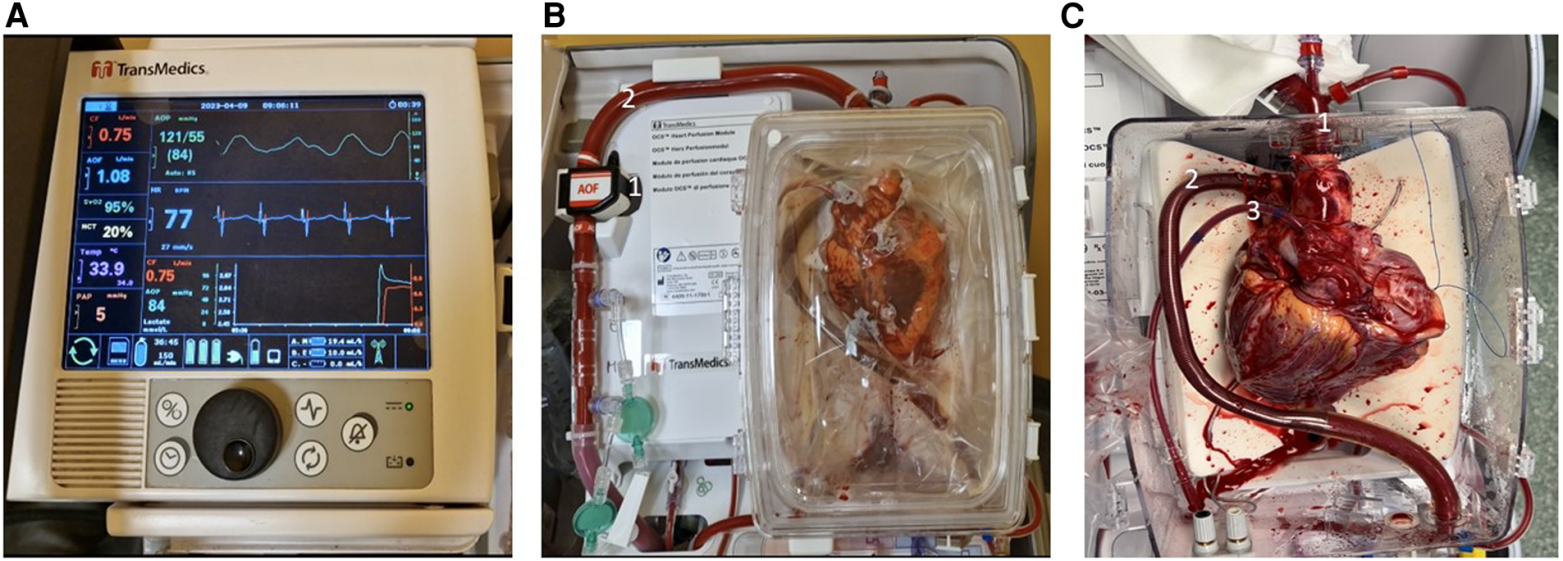
The transmedics organ care system. (**A**) Wireless monitor/controller. (**B**) Heart perfusion module: (1) aortic flow probe; (2) aortic perfusion line. (**C**) Instrumented heart: (1) aortic connector; (2) pulmonary artery cannula; (3) left ventricular venting tube.

The PSP device consists of two canisters, one internal and one external (Figure 1A). The internal canister is filled with cold storage saline solution (4°C–8°C), and the donor heart is submerged into it, after being connected to the canister lid by means of an aortic connector (Figure 1B). The most widely used solutions for heart preservation are the Celsior, the University of Wisconsin (UW) and the Custodiol (histidine-tryptophan-ketoglutarate—HTK). Then, the inner canister is placed into the outer one, creating an insulating air chamber, and outside this system is surrounded by single-use cooling ice packs. A thermometer connected with the internal canister allows continuous monitoring of the temperature (Figure 1C).

The GUARDIAN study is a post-market, observational registry of adult and pediatric patients who received a donor heart preserved and transported using either the PSP or standard preservation methods. Using data of 877 patents enrolled in the Guardian heart registry by 16 US centers, two cohorts of 249 patients were propensity matched according to the technique of graft preservation. Although the 1-year survival did not statistically differ between the two cohorts (*p* = 0.12), PSP preservation significantly reduced severe PGD rate, compared to ice-cold storage (4% vs. 10%, *p* = 0.01) (10). The use of PSP has also proved to be cost-beneficial. In a recent study that compared two groups of 87 matched patients (PSP and ice-cold storage), post-HTx costs were significantly lower when donor organs were preserved with PSP. This figure reflected a significant role of PSP in reducing incidences of severe PGD (5.7% vs. 16.1%, *p* = 0.03) and employment of mechanical circulatory support after HTx (21.8% vs. 40.2%, *p* = 0.009), and thus the recipient hospital length of stay (11).

Histological analyses of myocardial biopsies taken as soon as the donor hearts were reperfused during HTx, showed that grafts preserved with PSP appeared to have less interstitial edema and myocyte damage compared to those preserved with traditional ice storage (12).

## 3. Machine perfusion systems

Differently from static cold storage, MP systems represent dynamic methods of preservation that prevent extra ischemic time and thus potentially provide better preservation of the donor heart. Two types of MP systems are currently used in HTx: hypothermic (HMP) and normothermic (NMP) machine perfusion. As reported below in this paper, NMP could also provide the opportunity to assess the metabolic and functional status of the donor graft.

However, MP generally expensive and require well-trained and specialized personnel to set up the devices and manage any complications and malfunctions, which could otherwise damage the donor organ. Unfortunately, these limitations hamper a routine adoption of MP, and therefore their extensive employment is made even more difficult in centers with a low volume of activity and with scarce economic resources.

### 3.1. Hypothermic MP

The rationale of HMP preservation consists in reducing the metabolic requirements of the heart with an optimal and homogeneous cooling (below 10°C), while providing continuous metabolic support though perfusion with oxygenated, nutrient-enriched medium to limit as much as possible intracellular anerobic metabolism and consequent acidosis. Experimental studies performed on large animal models have suggested that compared to SCS, HMP could attenuate tissue injuries and provide superior myocardial function after HTx (13); nevertheless, clinical adoption of HMP has been limited due to the concerns about a reliable functional assessment of this system. Another major concern is related to the risk of edema during HMP preservation and after reperfusion. However, using a perfusate of high osmotic and/or oncotic power at low perfusion pressures could prevent edema formation. Moreover, edema related to HMP employment was reported to be more likely interstitial and reversible, with limited impact on post-HTx cardiac function (14).

Three single-center clinical studies have so far analyzed the effects of HMP using three different perfusion solutions: Wicomb et al. in 1984 used crystalloid cardioplegic solution in 4 patients (15), Hill et al. in 1997 used colloid cardioplegic solution in 8 patients (16), and more recently, in 2020, Nilsson et al. reported their experience employing a home-made MP with hyper-oncotic cardioplegic solution supplemented with hormones and erythrocytes, so called “non-ischemic hypothermic perfusion” (NIHP) (17). In their series, 6 patients who received donor hearts preserved with NIHP showed better outcomes 6 months after HTx compared to 25 recipients who received SCS preserved grafts (100% vs. 84% survival rate). Based on these preliminary promising results, the Xvivo Perfusion AB (Goteborg, Sweden) has patented the NIHP and further developed it to a commercially available device; currently, a randomized clinical trial is ongoing to assess patient and graft survival comparing NIHP to SCS (18).

The Lifecradle® Heart Preservation System is a HMP, currently under development, that uses hypothermic, oxygenated, nutrient perfusion at 5°C, in a controlled and monitored environment. The safety and efficacy of this device will be defined on the basis of clinical evidence, currently pending.

#### 3.1.1. XVIVO perfusion

The XVIVO Heart Perfusion System consists of a roller pump, an oxygenator, a leukocyte filter and a cooler/heater. After cardiectomy, the donor heart is connected to the XVIVO device with an aortic cannula. Then, it is submerged into the reservoir, filled with 2.5 L of perfusion solution to which 500 ml of donor and recipient immunologically-compatible irradiated blood are added. The oxygenated perfusion solution (with a hematocrit of about 15%) is pumped into the aortic root to maintain the pressure of 20 mmHg to provide a coronary blood flow between 150 and 200 ml/min in a non-beating heart state. The temperature is constantly maintained at 8°C and the pH at 7.4 value. During transportation the XVIVO device does not need continuous monitoring and power source.

The initial Australian experience on 13 patients with the Xvivo NIHP for HTx showed promising results. In fact, there was no post-operative mortality and only 1 patient required veno-arterial extracorporeal membrane oxygenation (ECMO) due to secondary graft failure. The authors reported a median donor graft ischemic time of 404 min but since the period of non-ischemic perfusion was included this data could be misleading (19).

The XVIVO innovative technology was employed for xenograft preservation during the modified pig-to-human cardiac xenotransplantation performed at the Maryland University on January 2022 (20).

### 3.2. Normothermic MP

Normothermic MP systems perfuse the heart with oxygenated blood and enriched solutions, keeping it beating and at a near-physiological temperature of about 34°C. Currently the Organ Care System (OCS, TransMedics Inc, Andover, MA) represents the only NMP system commercially available for clinical use in HTx. *Ex vivo* perfusion with this device is particularly attractive when “extended criteria” for donor organs procurement have to be further evaluated; this system, besides limiting graft ischemic time, allows a real-time monitoring of the donor graft assessing hemodynamic parameters and lactate concentration, the latter being the main marker of organ metabolism, with a timely identification of potentially unsuitable grafts. Moreover, for these reasons, OCS is increasingly employed in resuscitation and assessment of organs from donation after circulatory death (DCD).

#### 3.2.1. The organ care system

**Figure 2:**
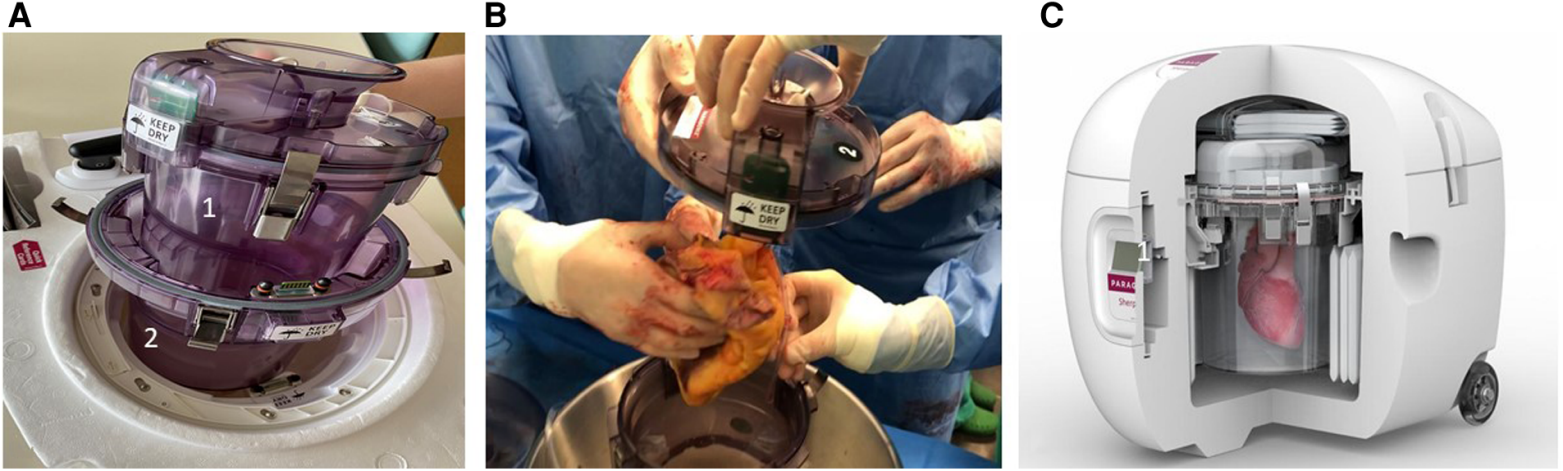
The paragonix sherpa-Pak system. (**A**) Internal (1) and external (2) canisters of Paragonix Sherpa-Pak. (**B**) The donor heart connected to the internal canister lid. (**C**) Overview of the system: (1) display and bluetooth data transmission module.

The OCS consists of a portable platform and is composed of a wireless monitor/controller (Figure 2A) and a circuit in which the donor blood perfuses the beating and empty donor heart (Figure 2B). The donor blood is mixed with a specific priming solution which contain mainly mannitol, electrolytes, vitamins and antibiotics; during *ex vivo* perfusion two other solutions are infused into the circuit: the catecholamine solution, containing epinephrine to replenish the depleted catecholamine level and the maintenance solution enriched with adenosine, aiming to modulate the coronary artery resistance.

The donor blood is oxygenated and maintained at 34°C by a heater-membrane oxygenator module, and it is delivered into the aortic inflow cannula by a peristaltic pump, after both venae cavae are closed. The blood perfuses the coronary vessels, reaches the coronary sinus and eventually the pulmonary artery, where an outflow cannula collects it closing the perfusion circuit (Figure 2C). The blood that does not reach the coronary sinus (because of aortic regurgitation and bleeding from cut surfaces), is collected and re-infused into the circuit. Flow and pressure of the blood are registered by probes. Stopcocks permit sampling of blood for arterial and venous lactate concentration monitoring. Coronary resistance, arterial pressure and coronary flow can be modified by acting on the pump flow speed and/or the maintenance solution infusion rate. OCS settings are adjusted to keep the mean aortic pressure between 80 and 100 mmHg, and coronary blood flow between 700 and 900 ml/min. Graft function is assessed by continuous monitoring of aortic pressure, coronary flow and the total arterial and differential artero-venous lactate profile. An arterial lactate level >5 mmol/L is considered an index of myocardial damage and thus a contraindication to use the graft, as well as an unfavorable artero-venous lactate production pattern as evidenced by a venous lactate concentration higher than arterial lactate level. The OCS device can be transported either by car, plane, or helicopter.

## 4. NMP employment

During the last decade, normothermic *ex vivo* perfusion has emerged as a key factor in expanding the cardiac donor pool, as it favors a safer employment of donor hearts selected using extended criteria by limiting ischemic time and allowing graft assessment. The following are some of the advantages of using NMP.

### 4.1. To shorten the ischemic time

The continuous coronary perfusion by means of oxygenated enriched blood is the main advantage of normothermic machine perfusion. The results of the PROCEED II controlled trial demonstrated the non-inferiority of the NMP compared to the traditional ICS. Although patient and graft survival between both study arms were similar, the OCS group reported a significantly shorter graft ischemic time. Interestingly, the 4 hearts of OCS group that were discarded because of an increasing lactate concentration, after histological analysis revealed signs of infarction, contusion and severe unrecognized left ventricular hypertrophy (21).

The OCS could be an effective tool to ensure graft quality when the expected graft ischemic time exceed the traditional “safe threshold” of 4 h, favoring long-distance organ retrieval. Two case reports presented successful HTx after preservation times of a donor heart as long as 10 h (22) and 16 h (23). The relatively safe non-ischemic “out of body time” could also be advantageous in particular situations, such as when an unexpected finding is discovered during organ retrieval that needs a histological definition (24).

In Table 1 are reported graft ischemic and “out of body” time data derived from single-center and retrospective studies (25–31) when grafts were preserved with OCS.

**Table 1:** Studies of normothermic machine perfusion for hearts from DBD with and without ICS as control group.

Author	OCS group					ICS group			
	no. of pts	Donor age (range)	Recipient age (range)	Out of body time (min)	Graft ischemic time (min)	no. of pts	Donor age (range)	Recipient age (range)	Graft ischemic time (min)
Ardehali et al. (21)	67	35 (18–58)	56 (20–75)	324 ± 79	113 ± 27	63	34 (13–60)	57 (20–76)	195 ± 65
Garcia Saez et al. (25)	26	37 ± 12	43 ± 13	371 ± 102	87 ± 15	–	–	–	–
Kaliyev et al. (26)	13	43 ± 15.5	40 ± 12	330.3	83 ± 8	–	–	–	–
Koerner et al. (27)	29	36 (17–54)	50 (37–64)	297	52	130	–	50.7 (37–64)	–
Sponga et al. (28)	14	46 ± 11	64 (35–75)	452	132 ± 28	24	44 ± 13	57 (30–73)	225 ± 48
Sponga et al. (29)	21	47 ± 11	58 (24–66)	272 ± 65	145 ± 29	79	48 ± 13	60 (28–73)	213 ± 63
Sato et al. (30)	16	–	52 ± 15.5	362 ± 153	114 ± 51	18	–	59 ± 16	183 ± 34
Rojas et al. (31)	68	37	49 ± 13	381 ± 74	115 ± 43	51	44.5	59 ± 13	228 ± 43

DBD, donation after brain death; OCS, organ care system; ICS, ice-cold storage.

**Table 2:** Characteristics of current preservation techniques for cardiac grafts.

Technique	Cost	Temperature control	Reduction of IT	Function assessment	Expertise/complexity	Graft manipulation
ICS	+	+	−	−	+	−
PSP	++	+++	−	−	+	−
OCS	+++	+++	+++	++	+++	+
NIHP	+++	+++	++	−	++	NA

IT, ischemic time; ICS, ice cold storage; PSP, Paragonix SherpaPak; OCS, organ care system; NIHP, non-ischemic hypothermic perfusion; NA, not available.

### 4.2. To assess organ adequacy

NMP has shown interesting and promising results when utilized in extended-criteria DBD donors, and DCD. Extended-criteria were generally defined according to these parameters: >50 years of age, a history of drug abuse, cardiac resuscitation, coronary artery disease (CAD), expected graft ischemia time >4 h, left ventricular ejection fraction (LVEF) <50%, or interventricular septum thickness (IVS) >14 mm.

Data regarding marginal DBD donors are derived mainly from single-center observational studies. A previous report from our group demonstrated that NMP, compared to ICS in extended-criteria donor hearts, seemed to provide more stable hemodynamic conditions after HTx, reducing complications and allowing optimal outcomes. In fact, 5-year survival of OCS-preserved-heart group was 100% vs. 73% of CS control group (*p* = 0.04). These results are also supported by histopathological and ultrastructural evidence, suggesting better myocardial preservation in NMP grafts (28).

The EXPAND trial, which was designed as a single-arm multicenter study, evaluated the impact of OCS preservation in extended-criteria donor hearts. Out of 93 donor hearts evaluated, 75 were utilized for HTx (81% utilization rate). The 30-day post-HTx survival rate was 94.6% and the incidence of severe PGD in the first 24 h was 10.7%. Moderate to severe PGD was observed in 14.7% of patients (32).

In our experience, out of 74 grafts preserved with NMP, a total of 9 grafts were discarded (88% utilization rate) due to a progressive increase in lactate concentration, expression in most cases of severe left ventricle hypertrophy, scarring and undiagnosed coronary artery disease. In one case, NMP real-time evaluation of lactate trend permitted to discard an apparently adequate organ which, at gross pathological examination, revealed a dissection of the right coronary artery at 4 mm from its origin (33). Considering organ assessment and expansion of donor pool, interestingly at our center a donor heart with a myocardial bridge, which should be a relative contraindication to Htx, was successfully and safely transplanted in a 66-year-old recipient. This was possible owing to the continuous evaluation of cardiac function, which allowed to consider such graft suitable for HTx (34).

In case of DCD, the heart is exposed to prolonged periods of warm ischemia and to right atrial and ventricular over-distension during cardiocirculatory arrest, with possible irreversible myocardial injury. Thus, a post-asystolic functional assessment is of paramount importance when evaluating these hearts. In clinical practice, DCD hearts are retrieved with either direct procurement and perfusion (DPP) or normothermic regional perfusion (NRP). In DPP, the heart is removed after confirmation of death and expeditiously reperfused using the OCS (35–38). Instead, in NRP the employment of ECMO or cardiopulmonary bypass (CPB) facilitates cardiac resuscitation. After the donor is weaned from circulatory support, the heart is assessed “*in situ*” and if adequate recovery is observed it is retrieved and preserved with OCS or ICS (39, 40).

The introduction of NMP in clinical practice has permitted to utilize DCD donor hearts with gratifying results (35–40). Furthermore, when compared with the current “gold standard” ICS-preserved DBD hearts, the OCS-preserved DCD grafts have shown to provide comparable results (36, 40). A recent randomized controlled trial compared the outcomes of 90 HTx using DCD hearts reanimated, preserved and assessed with the OCS with that of 90 HTx perfomed by using DBD hearts preserved with ICS. The use of OCS resulted in a high rate of graft utilization rate (89%) in DCD group, and criteria for graft non-use were rising lactate concentrations, visual contractility anomalies or both. The 6 months risk-adjusted survival of HTx from DCD grafts (94%) was noninferior to that after transplantation of DBD hearts (90%). However, the rate of severe PDG was higher in HTx from DCD (15%) vs. DBD (5%) grafts (41).

The method of retrieval (DPP or NRP) was not associated with different outcomes after HTx according to the results reported in the experience of Messer et al. (40).

### 4.3. To facilitate HTx in high-risk patients

Heart MP could also play a protective role in high-risk recipients, particularly in those supported by durable mechanical circulatory support or who have undergone previous complex operations (25, 29, 31). HTx in these patients might be technically demanding and often requires a tedious dissection and prolonged CPB to complete the removal of intrathoracic ventricular assist devices or the isolation of the cardiac structures. The use of MP allows optimization of coordination between retrieval and implanting teams, favoring a meticulous and stress-free preparation of the recipients while the donor graft remains perfused. Moreover, this might reduce post-procedural bleeding and transfusions of blood products with improvement of hemodynamic stability after HTx. In a previous report from our group, in a series of patients bridged to HTx with MCS, OCS perfusion conferred a protective role regarding PGD development after HTx, compared to CS (7% vs. 42%, *p* = 0.03) (29).

### 4.4. To recover the injured graft

Sarcomere changes, such as Z-line thickening and/or non-orthodox banding were reported in donor hearts immediately after retrieval (28). After *in situ* reperfusion during HTx, hearts preserved with ICS are frequently affected by myocardial injury, with damage of contractile myofilaments and organelles, including mitochondria. The *ex situ* perfusion with OCS is reported to be effective in reconditioning donor hearts, that are maintained metabolically active and able to heal ultrastructure changes (28, 42).

Donor hearts, selected according to expanded-criteria, appear to be best treated by NMP, especially when severe hypotension or cardiac arrest occurs during the retrieval phase and since this technique could hamper the negative effect of cold storage on ultracellular cardiac function (42).

## 5. Future perspectives

MP systems could be useful platforms for cardiac conditioning before transplantation, since they create a “safe period” between procurement and transplantation during which the organ could potentially be manipulated. Graft immunomodulation, via infusion of viral vectors (43, 44) or mesenchymal stem cells (MSC) injection (45), could modify its immunogenic capacity and reactivity.

In HTx, donor infusion of MSCs has been shown to prolong the survival of a semi-allogeneic HTx in a mouse model through the generation of regulatory T cells (46). In addition to MSC, also the injection of extracellular vesicles secreted from cardiomyocytes (iCM-EVs) derived from induced pluripotent stem cells have been demonstrated to lead to functional recovery hearts injured from pathologic hypertrophy. Since their content is mainly composed of miRNAs that modulate specific cardiac processes, they could represent a promising cell free alternative for cardiac recovery (47). MP could also represent the ideal platform for the introduction of viable and competent mitochondria into the graft tissue prior to reperfusion to improve the heart metabolic function and to reduce the ischemia-reperfusion injury (48). Preliminary scientific reports, confirming the potential for clinical application of these techniques, underline the need for prolonged graft manipulation in order to achieve a significant effect, making NMP an irreplaceable method (43–49).

In an effort to further suppress tissue metabolism and thus increase a safe preservation duration, sub-zero preservation techniques have been investigated in preclinical studies (50). Isochoric supercooling, that limits the cristallization of ice by controlling temperature and volume systems, and vitrification, that involves a large amount of cryopreservation and a rapid cooling scheme, are two intriguing techniques for “freezing biological time”. Despite experimentary results on cells, tissues and small-animal organs are encouraging, successful employment to larger-volume organs remains to be demonstrated (51).

Various pharmacological agents have also been investigated in order to better preserve the graft by interfere with the ischemia-reperfusion injury mechanism. Donor simvastatin treatment might significantly improve graft function after transplantation (52), while valproic acid seems to stimulate cardioprotective immune-metabolomic pathways (53).

Interfering with the immune system during graft preservation could lead to a smooth immunological response of the recipient against the organ, and thus reduce the degree and number of rejections. Antioxidative agents, and inhibitors of cytokines production and activity and maturation of lymphocytes, as well as inhibitors and modulators of cellular receptors involved in signalling pathways and adhesion molecules expression are described as intriguing potential treatments (54).

## 6. Conclusions

The field of graft preservation is subject of notable innovations. ICS was the standard technique for 50 years, but it does not allow temperature monitoring and exposes the heart to freezing damage. Increasingly, the use of ICS is being replaced with controlled hypothermic preservation using the Paragonix SherpaPak™ device, that in preliminary reports seems to offer advantages over ICS in therms of better organ preservation and clinical outcomes. Paragonix SherpaPak™ transport system has all the premises to be considered the near-future standard for donor heart cold storage, being able to allow temperature control, avoid tissue freezing, be relatively cheap and simple to use.

On the other hand, MP systems can represent the opportunity to assess and recondition the donor heart and are increasingly employed worldwide in an attempt to expand the donor pool for HTx. However, despite interesting results, the role of MP in HTx remains still debatable, mainly because of higher costs and training needs than those required for CS.

At present, while the clinical effectiveness of HMP has to be investigated, NMP with the OCS seems to allow safe utilization of DCD and extended-criteria donor organs, combining two major advantages: to limit the graft ischemic time and to verify cardiac function by direct visual inspection and through assessment of metabolic values, and haemodynamic parameters. Maintenance of myocardial aerobic metabolism during preservation could lead to better donor heart quality compared to traditional CS. Thus, the NMP represents an effective technique that permits to expand the donor pool, allowing acceptance of grafts which would have otherwise been refused, while maintaining satisfactory safety levels. In fact, NMP allows to identify unsuitable grafts and discard them before transplantation, reducing the risk of PGD and its life-threatening sequelae (20, 27, 31).

Some issues related to MP systems should be more thoroughly investigated in the near future to further improve this technique, such as additional metabolic support, solution components and optimal perfusion settings. Also, the identification of other functional parameters or biomarkers, apart from lactate levels, could be of paramount importance to increase the sensitivity of MP to help clinicians in assessing suitability of perfused donor grafts.

The main drawback of NMP is that it requires an experienced and well-trained professional team, to manage the interaction between the donor organ and the *ex vivo* perfusion, and to promptly intervene in case of machine malfunction or user error. In fact, since the donor heart is preserved in a beating normothermic state, the margin of safety is limited in case of complications or non-appropriate NMP management due to the risk of catastrophic and irreversible warm ischemia of the graft (Table 2).

In conclusion, the satisfactory results reported in HTx with high-risk recipients and extended-criteria donors highlight the effectiveness of NMP in complex cases, particularly in unfavorable combinations of donor, procedural and recipient characteristics. The results of ongoing multicenter clinical trials investigating on heart MP are required to confirm these expectations.
